# Global and local selection acting on the pathogen *Stenotrophomonas maltophilia* in the human lung

**DOI:** 10.1038/ncomms14078

**Published:** 2017-01-19

**Authors:** Hattie Chung, Tami D. Lieberman, Sara O. Vargas, Kelly B. Flett, Alexander J. McAdam, Gregory P. Priebe, Roy Kishony

**Affiliations:** 1 Department of Systems Biology, Harvard Medical School, Boston, Massachusetts 02115, USA; 2 Department of Pathology, Boston Children's Hospital, Boston, Massachusetts 02115, USA; 3 Division of Infectious Diseases, Department of Medicine, Boston Children's Hospital, Boston, Massachusetts 02115, USA; 4 Department of Laboratory Medicine, Boston Children's Hospital, Boston, Massachusetts 02115, USA; 5 Division of Critical Care Medicine, Department of Anesthesiology, Perioperative and Pain Medicine, Boston Children's Hospital, Boston, Massachusetts 02115, USA; 6 Faculty of Biology, Technion Israel Institute of Technology, Haifa 32000, Israel; 7 Faculty of Computer Science, Technion Israel Institute of Technology, Haifa 32000, Israel

## Abstract

Bacterial populations diversify during infection into distinct subpopulations that coexist within the human body. Yet, it is unknown to what extent subpopulations adapt to location-specific selective pressures as they migrate and evolve across space. Here we identify bacterial genes under local and global selection by testing for spatial co-occurrence of adaptive mutations. We sequence 552 genomes of the pathogen *Stenotrophomonas maltophilia* across 23 sites of the lungs from a patient with cystic fibrosis. We show that although genetically close isolates colocalize in space, distant lineages with distinct phenotypes separated by adaptive mutations spread throughout the lung, suggesting global selective pressures. Yet, for one gene (a distant homologue of the *merC* gene implicated in metal resistance), mutations arising independently in two lineages colocalize in space, providing evidence for location-specific selection. Our work presents a general framework for understanding how selection acts upon a pathogen that colonizes and evolves across the complex environment of the human body.

## Abstract (web summary)

The authors sequence the genomes of 552 bacterial isolates sampled across 23 sites of the lungs of a patient with cystic fibrosis, and identify bacterial genes under global and location-specific adaptation.

Bacterial species evolve in the human body, whether within the microbiome^1^,^2^,^3^,^4^ or over the course of an infection^5^,^6^,^7^. Mutations can sweep through the population^8^ or lead to diversification into distinct subpopulations that differ genotypically and phenotypically^5^,^6^,^7^,^9^,^10^,^11^,^12^,^13^,^14^,^15^. Subpopulations within an individual can be spatially segregated across the structured environment of the human body^14^,^16^,^17^,^18^,^19^, but it is unknown to what extent such spatial variations reflect local differences in selective pressures. Separating which genes are important for the local versus global adaptation of a pathogen is critical for predicting and possibly manipulating the survival strategy of the pathogen. However, it remains challenging to determine whether a spatially enriched genotype is the result of local selection or physical isolation and drift^14^.

Here, to disentangle these confounding effects and identify local selection, we ask whether independently occurring adaptive mutations colocalize in space. We sample and sequence the whole genomes of 552 *Stenotrophomonas maltophilia* isolates from 23 sites across the explanted lungs of a cystic fibrosis (CF) patient; *S. maltophilia* is a Gram-negative bacillus and an emerging pathogen in CF as well as in nosocomial and community-acquired infections^20^. We first determine specific genes that were under selection during diversification by identifying genes with recurrent mutations. We then test whether mutations of a gene under selection that occurred independently in different lineages colocalize to the same set of sites despite sufficient time for dispersal. This approach therefore allows us to separate global and local selective pressures acting at the level of genes in pathogen populations.

## Results

### Sampling *S. maltophilia* diversity from lung tissue sites

**Figure 1:**
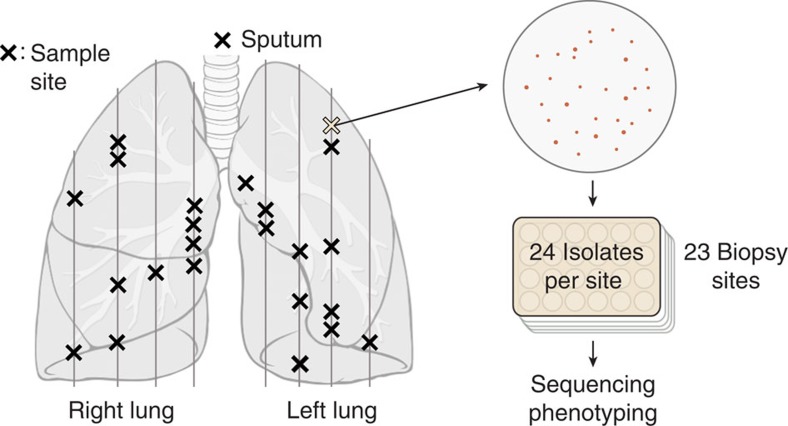
Sampling *Stenotrophomonas maltophilia* populations across explanted CF lungs. From each lung, we took 4 parasagittal cross-sections along the medial to lateral axis. Each sample was homogenized and plated on MacConkey agar; 24 isolates were randomly selected from 23 tissue samples that exhibited growth. All isolates were whole-genome sequenced and phenotyped. Sample site locations are approximate. Lung figure adapted from original by Patrick J. Lynch and C. Carl Jaffe (goo.gl/iC8AjM), CC-BY-2.5.

We studied the population diversity of *S. maltophilia* within and between multiple tissue sites sampled across the lungs of a CF patient. The patient was chronically infected with *S. maltophilia* and underwent bilateral lung transplantation. *S. maltophilia* was first detected in the patient 3 years before the transplant and had been the dominant pathogen in the lung. Sputum was sampled from the patient 1 day before the transplant. Hours after the lungs were removed from the patient, they were each cut parasagittally into four cross-sections; the knife was sterilized between slices to minimize contamination. Tissue specimens (31 total, 0.1–1 cm per side) were sampled from different sites in each section (Fig. 1 and Supplementary Table 1). Examination of corresponding histologic sections of tissue specimens confirmed a diversity of anatomic sites (Supplementary Fig. 1). Specimens and sputum were homogenized and plated for single colonies, and 24 *S. maltophilia* isolates were randomly chosen from each sample that exhibited bacterial growth (23 specimens and sputum; total 576 isolates).

### Population diversified into multiple coexisting lineages

Whole-genome sequencing and phylogenetic analysis revealed that the pathogen diversified into multiple coexisting lineages. Sequencing the whole genomes of all isolates revealed 334 polymorphic genomic positions (Supplementary Data 1). A parsimony tree exposed several coexisting lineages, including two major lineages A and S, with lineage S exhibiting a small colony variant morphology (Supplementary Fig. 2), and two minor lineages B and C. These lineages were separated by 47 single-nucleotide polymorphisms (SNPs) and had copy number variations in 4 gene regions (Supplementary Fig. 3). However, lineages had very similar genomic architecture (Supplementary Fig. 4) and the extent of population diversity was consistent with the expected, assuming mutations accumulating during the infection period with a molecular clock typical of CF pathogens^21^. Therefore, similar to other pathogens in chronic infections^6^,^12^,^13^,^15^,^16^, these results indicated *de novo* diversification of a single clone into multiple coexisting lineages within the patient.

### Identifying genes undergoing adaptive evolution

**Figure 2:**
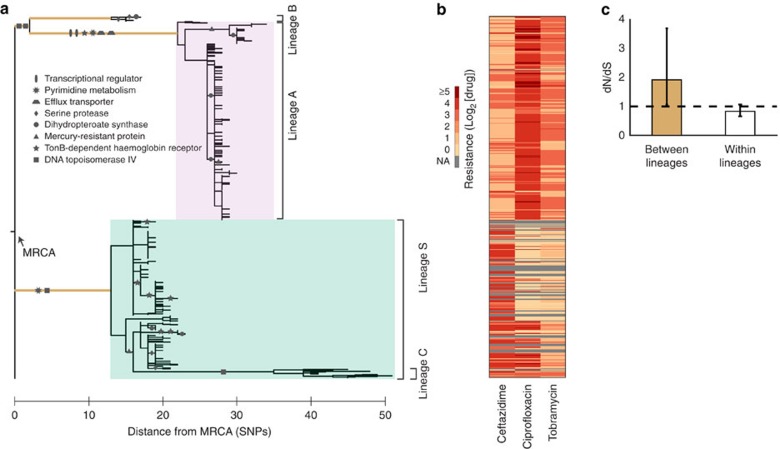
Positive selection drove differentiation of *S. maltophilia* into distinct coexisting lineages within the patient. (**a**) Parsimony tree of the population constructed from 334 polymorphic mutations. Most recent common ancestor (MRCA) on the left. The mutations of eight genes with recurrent mutations are indicated on the tree, each gene with its own symbol. Lineages A and S (small colony variant) form the majority of the population. Minor lineages B and C are also indicated. (**b**) Resistance profile of all isolates against three antibiotics used in treatment, in twofold drug concentrations. Each row is an isolate aligned to its position on the phylogeny. NA, not available; 0, isolate grew only on no drug plates. The differences in resistance between lineages A and S were highly significant: ceftazidime *P*=4 × 10^−39^, ciprofloxacin *P*=3 × 10^−43^, tobramycin *P*=2 × 10^−34^, Kolmogorov–Smirnov test. (**c**) Lineage-separating mutations are under positive selection (green; dN/dS=1.9, *P*=0.027, 95% confidence interval (CI) 1.06–3.70), whereas within-lineage mutations are neutral (white; dN/dS=0.93). Error bars indicate 95% CIs.

Lineage differentiation was driven by adaptive selection. Mutations separating lineages showed elevated ratio of nonsynonymous to synonymous substitutions (dN/dS=1.9, *P*=0.027; Fig. 2c), indicating a signal for positive selection consistent with previous findings in other CF pathogen, *Pseudomonas aeruginosa*^16^,^22^. Many of these lineage-separating mutations appear in genes known to be important for virulence (Supplementary Table 2): for example, one mutation in a secreted serine protease (StmPr2/AprV2 homologue), recently shown to contribute to toxicity in human epithelial cells as well as degradation of extracellular matrix protein and interleukin-8 (ref. 23), could disrupt a stabilizing disulfide bridge in a disordered region of the protein (Supplementary Fig. 5). In addition to the dN/dS signal, which may be biased because of hitchhiking, we found further support for positive selection by detecting four genes with at least two lineage-separating mutations (Supplementary Fig. 6a and Supplementary Table 2); mutational events in these genes are marked in Fig. 2a. As two of these genes are implicated in antibiotic resistance (DNA topoisomerase IV and an efflux transporter), we measured the resistance of every isolate to three different drug classes administered to the patient early in the infection. Indeed, there was a significant difference in resistance profiles between the lineages (Fig. 2b). Altogether, the signal for positive selection from dN/dS, genes with recurrent mutations and difference in resistance phenotypes support that adaptive selection drove lineage divergence.

Evidence of adaptive evolution was also observed within the lineages. Although mutations within lineages did not show a dN/dS signal for selection as a whole (dN/dS=0.93, 95% confidence interval 0.74–1.20), four genes were mutated at least three or more times, much more than expected by chance (Supplementary Fig. 6b; genes labelled in Fig. 2a, listed in Supplementary Table 3). This suggests positive selection acting upon these genes (*P*=0.004, a threshold of 3 mutations was chosen to best remove false positives, bootstrapping a random allocation of mutations across the genome; see Methods). The four genes encode a mercury resistance protein homologue (*merC* homologue), dihydropteroate synthase, a TonB-dependent haem/haemoglobin receptor (*shuA* homologue) and a serine protease (*mucD/htrA/degP* homologue). Indeed, all 15 mutations across these multiply mutated genes were nonsynonymous, and many mutations were found clustered together near functional domains of the protein (Supplementary Fig. 7); furthermore, the *shuA* homologue was mutated 6 times within lineage S, but none within lineage A, the ancestor of which carries a mutation in this gene. Together, these results show that different selective pressures can act on the two major lineages during their separation and later on during their diversification.

### Major lineages are distributed across most sites

**Figure 3:**
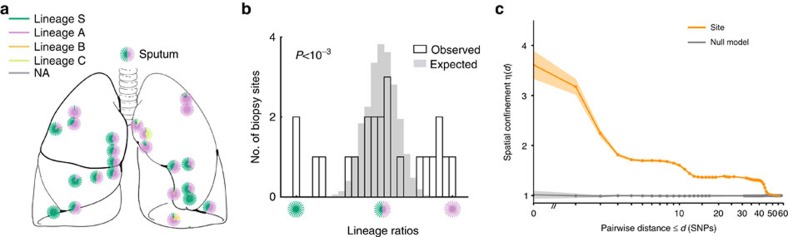
Mapping the spatial distribution of lineages and estimating the dispersion rate of a mutation. (**a**) The approximate location of each tissue sample is labelled on the lung. Each radial line is coloured by the lineage membership of an isolate with the exception of those that were undetermined because of inadequate sequencing coverage (grey). The sputum population is shown at top. Lung figure adapted from original by Patrick J. Lynch and C. Carl Jaffe (goo.gl/iC8AjM), CC-BY-2.5. (**b**) The observed ratios between lineage A and S across sites are significantly different from the expectation in a well-mixed environment (*P*<10^−3^, χ^2^ test). (**c**) We calculated the likelihood *η(d)* that pairs of isolates separated by *d* or less SNPs are in the same site (orange line); grey line indicates null model. Shaded error bars indicate 1 s.d. with respect to null model. We inferred a dispersion time of *d*∼3.

Mapping the spatial distribution of the major lineages revealed that they were not segregated between the two lungs and not even between lobes or sites. Lineages coexisted in most sites, indicating that they dispersed throughout most of the lungs over the 3-year infection (Fig. 3a). Notably, both major lineages but neither minor lineage were represented in the sputum sample, suggesting that clinical samples capture only the gross-level diversity of *S. maltophilia*. Similar to previous results obtained for *P. aeruginosa*^14^, the ratio between major lineages A and S across sites significantly deviated from the expectation under a well-mixed model (Fig. 3b; *P*<10^−3^, χ^2^ test). Indeed, some sites were dominated or even purely occupied by only one of the two major lineages; minor lineages B and C were also localized to particular sites. Principal component analysis of β-diversity between site populations via Unifrac^24^ did not show any site clustering by lung location (Supplementary Fig. 8), though lineage-enriched sites may be more likely to be found in the peripheral planes of the lung (Supplementary Fig. 9). Although contamination during sampling is possible, there was no evidence that parasagittal slicing of the lungs mixed site populations (Supplementary Fig. 8). Altogether, major lineages dispersed throughout most of the lungs, yet were significantly enriched in some sites.

### New mutations disperse rapidly out of a site

Spatial confinement of alleles increased towards younger branches of the phylogeny, providing an estimate of their rate of dispersal. As a new mutation arises, we expect that it will first be confined to its site of origin and gradually spread to other sites, a phenomenon known as ‘isolation by distance'^25^,^26^,^27^. Following Croucher *et al*.^28^, we defined spatial confinement *η* as a function of mutational age *d* by the fraction of all isolate pairs separated by a genetic distance of *d* or less SNPs that were in the same site, normalized by the expected fraction under a null model where isolates are randomly distributed (*η*=1 when there is no spatial confinement; see Methods)^28^. As expected, spatial confinement was high for genetically similar isolates (Fig. 3c, orange line; *η*>1 for *d<*3, *P*<10^−3^). Colocalization was rapidly lost as the genetic distance increased, suggesting that a new mutation disperses out of a site in the time it takes to accumulate only ∼3 SNPs, translating to ∼4.5 months from an estimate of the molecular clock (see Methods). This relatively rapid dispersion is consistent with the observation that *S. maltophilia* frequently resides in sputum-filled airways^29^,^30^. However, despite this potential for rapid dispersion, some genotypes maintained confinement longer than expected as evidenced by continued higher spatial confinement than the expectation in a null model (*η*>1 even at large *d*). This observation suggested regional isolation or local selection acting on a subset of mutations^14^.

### Identifying a gene under location-specific selection

**Figure 4:**
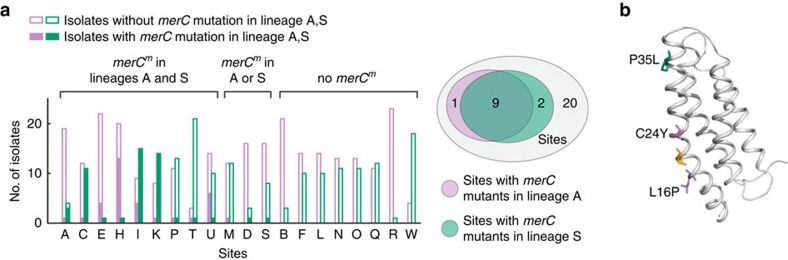
Location-specific selection of *merC* homologue mutants. (**a**) Independently occurring mutants of the distant *merC* homologue in both lineages A and S colocalize to the same sites (*P*=0.005, Fisher's exact test). (**b**) MerC is an inner membrane protein with four transmembrane domains^45^. The three mutational events in the *S. maltophilia* distant *merC* homologue were mapped to a protein model (generated via EVfold^31^,^32^). Mutation in lineage S (P35L) is highlighted in teal, whereas mutations in lineage A (C24Y, L16P) are highlighted in pink. The cysteine proposed to form a disulfide bond with C24 is highlighted in orange^45^ (C20); C24Y would disrupt this disulfide bond. All three mutations are found on the same side of the first transmembrane domain.

Location-specific selection was detected by asking whether independently occurring mutations in a gene under selection colocalized to the same sites. Adaptive lineages can be enriched in a site because of local selective pressures, yet physical segregation could also confine related genotypes to a location (Supplementary Fig. 9). To disentangle the signal for selection from this confounding factor, we focussed on the cases where independent mutations appeared in the same gene and asked whether they colocalized in space. Two genes were mutated in both lineages A and S: *merC* and dihydropteroate synthase. In one of these genes, a distant homologue of the *merC* gene (hereinafter referred to as *merC*), mutations that occurred independently in both lineages proliferated in the same set of sites (Fig. 4a; *P*=0.005, Fisher's exact test). Such location-specific expansion reveals that selection can sort mutations across space even under rapid dispersal. Sites with *merC* mutations were distributed throughout the lung, demonstrating that spatial selection can occur on a small length scale rather than at a gross physiological level such as a lobe or lung. Interestingly, mapping the mutations on a protein model of MerC (generated via EVfold^31^,^32^) revealed that they were on the same side of the first transmembrane domain (Fig. 4b), suggesting that a modification in this region, and not its absence, is important for survival in these sites. The *merC* gene has been associated with mercury resistance as well as mercury acquisition^33^ and found in the *mer* operon^34^,^35^, but in *S. maltophilia* and related species *merC* is found on the chromosome next to a cobalamin synthesis protein (P47K homologue) and near a Co-Zn-Cd metal resistance gene (Supplementary Fig. 10), supporting a divergent role for this homologue^35^. Metal resistance has been associated with increased antibiotic resistance^36^, and it is possible that *merC* is important for resistance to antibiotics such as ciprofloxacin (Supplementary Fig. 11). Altogether, the data show that selection for changing the *merC* homologue is location specific and that certain genotypes can locally proliferate because of site-specific selection.

## Discussion

In this work we set out to identify genes that are under location-specific selection by testing for colocalization of independently occurring mutations in the same gene. Although much of the adaptation seems to confer global selective advantage, we identified at least one gene apparently evolving under local selection. Such site-specific selection might contribute to long-term maintenance of diversity in chronic bacterial infections that could impede therapy. It is possible that additional spatial segregation may exist in length scales smaller than the resolution of our tissue samples. Furthermore, some spatial enrichment may be diminished by cross-contamination that could bias our dispersal rate estimator. We demonstrate how a static genotypic distribution of a population can be used to reveal its dynamics and migration rates. This approach can be used to understand location-specific selective forces, and more generally how genetic diversity of bacterial populations evolving in the human body is shaped by selection and migration in structured environments.

## Methods

### Harvesting tissue from explanted cystic fibrosis lungs

Before lung transplantation, the patient's parents provided informed consent for study of the excised lungs. The study was approved by the Boston Children's Hospital Institutional Review Board. At the time of transplant, the male patient, who has homozygous F508delta *CFTR* mutation, was 10 years old and had been treated for the 3 previous weeks with oral trimethoprim/sulfamethoxazole, oral linezolid and inhaled tobramycin. The patient had also received a 2-week course of intravenous meropenem, intravenous tobramycin, oral minocycline and oral linezolid 4–5 weeks before transplant. Similar courses of antibiotics had been administered 3–4 times per year over the 3 years before transplant. Excised lungs were immediately refrigerated until processed for sample collection that occurred ∼8 h following removal. In each of the left and right lung, four parasagittal cross-sections were made with a knife cleaned with ethanol between each slice. Each cross-section of the lung was laid down carefully on a sterile cutting board. Each individual tissue sample was procured with a separate sterile, disposable scalpel. Biopsy samples were placed in 15 ml Falcon tubes with 1 ml phosphate-buffered saline and immediately stored on ice. Adjacent to each site sampled for microbiologic culture, a second sample was procured for histologic examination.

### Histologic examination

Tissue sampled for histologic examination was fixed in formalin and processed for paraffin embedding. Paraffin-embedded tissue was sectioned and stained with haematoxylin and eosin, Gram and Steiner stains and examined via light microscopy (Supplementary Fig. 1).

### Sequencing *S. maltophilia* populations from biopsy sites

Each sample was homogenized using a tissue grinder (15 ml Covidien Precision Disposable Tissue Grinder) and frozen in 15% glycerol. The sputum samples were homogenized by incubation with 10 mM dithiothreitol and frozen in 15% glycerol. Frozen samples were thawed and plated with beads onto MacConkey II Agar (BD 221270) in 10-fold serial dilutions (10^0^ to 10^−4^). Of the 31 biopsies, 23 exhibited growth; the sputum sample also exhibited growth. For each biopsy/sputum with growth, we chose the dilution level at which we observed 50–300 colonies to ensure diversity while minimizing competition between strains; we then randomly picked 24 colonies into independent wells of 96 deep-well plates (Greiner Bio One 780271 Masterblock 96-well 2 ml sterile V-bottom) filled with 1 ml LB. Cultures were incubated overnight at 37 °C with shaking. Then, 150 μl of saturated cultures were frozen in 15% glycerol and stored at −80 °C, whereas the remaining volume was used to extract whole-genome DNA using Invitrogen PureLink Pro 96 Genomic DNA Purification Kit. Genomic libraries were prepared by diluting Illumina Nextera kit and using custom barcodes for multiplexing, as previously described^37^ and sequenced using paired-end 100 bp reads on the Illumina Hi-Seq 2000 platform.

### Determining polymorphic mutations from sequence data

FASTQ files were trimmed for adapter sequences with cutadapt^38^ v1.8.3 and filtered with sickle2550 (ref. 39) (quality threshold 25, length threshold 50). These reads were then aligned to the reference genome of *S. maltophilia* strain Ab55555 (GenBank accession ALOG01000000; 4,918,929 bp across 6 scaffolds) using bowtie2 (ref. 40) v2.2.4 (paired-end with maximum fragment length 2,000 bp, no-mixed, dovetail, very-sensitive, n-ceil 0, 0.01). All unaligned reads (∼10% per isolate) were pooled across the isolates and used to assemble contigs using velvet^41^ v1.2.10 with paired-end reads, a minimum contig length of 1,000 bp and coverage cutoff of 1,000. The resulting 107 contigs (containing 308,871 bp) and the Ab55555 genome were concatenated to create a new master reference genome. All trimmed and filtered reads were realigned to this master reference genome with the same parameters as above; the average alignment rate was 95%. In addition, 98% of isolates (565 of 576) had a coverage mode of 10 reads or higher (Supplementary Fig. 12) with an average mode value of 30, and 99% of isolates (570 of 576) had less than 8% unaligned reads. Only isolates with a coverage mode of >10 were used for the analysis, resulting in 565 isolates used for downstream analysis and 11 isolates being discarded.

For each isolate, we used SAMtools^42^ v1.3 to generate candidate mutated positions with respect to the reference genome (FQ <−30). We then combined these positions across all isolates and reduced the list to positions that were polymorphic within the population. Polymorphic positions were determined if any two isolates disagreed in the nucleotide calls with both calls having an FQ score of ≤−52. With this approached, we obtained 334 SNPs (7 of which were in the contigs). The raw calls for each read aligned at all 334 positions for the 565 isolates (576 sequenced minus 11 isolates without adequate coverage) were used to create a matrix of genotypes. An isolate without adequate coverage (defined as <4 reads) at the position were designated as N; if the difference between the major and minor allele frequencies were <60% of the major allele frequency, the position was also designated as N. In total, 63 entries from the 334 × 565 matrix of calls were designated as Ns.

### Phylogenetic analysis

All SNPs calls were concatenated as a string for each of the 565 isolates that met the coverage mode threshold to generate an input file for dnapars, part of the PHYLIP^43^ v3.696 package. We constructed a parsimony tree using the Ab55555 strain reference genome as the outgroup.

### Gene content

Using the coverage at each position of the reference genome given by the DP field of the VCF file, we determined the normalized coverage of each gene for each isolate. DP values are averaged across each coding sequence and divided by the coverage mode for each isolate, resulting in copy numbers in units of isolate mode. To detect genes with copy number changes, for each gene we calculated the difference between the highest and lowest copy number across isolates; a threshold was chosen to find genes with large differences (Supplementary Fig. 3a).

Of the 4,539 coding sequences in the *S. maltophilia* Ab55555 reference genome, 3,972 genes were present in the isolates in this study. Of these 3,972 genes, 47 genes exhibited a significant copy number difference between isolates (Supplementary Fig. 3b); their descriptions are in Supplementary Table 4. Genes that differed in copy number between the lineages included numerous phage proteins and a set of Type II secretion system proteins (Supplementary Fig. 3c).

### Calculating dN/dS

Each mutation was determined as nonsynonymous or synonymous according to the annotation of open reading frames in the GenBank file. To calculate the expected value of nonsynonymous to synonymous substitutions in the genome, all intragenic mutations among the lineage-separating SNPs were redistributed across the reference genome in intragenic regions that had sequencing reads; the type of mutation was also preserved (transitions versus transversions). The expected N/S was calculated as the average ratio of nonsynonymous to synonymous substitutions across 1,000 simulations, each conducted for the subset of lineage-separating mutations and the within-lineage mutations. The distribution from this null model was used to calculate the 95% confidence interval. The dN/dS was then calculated as the observed value of N/S divided by its expectation.

### Multiply-mutated genes

We created a null model for observing *g* multiply-mutated genes by randomly distributing the observed mutations across the genome that had coverage among the isolates (genomic positions that had a mean copy number of 1 across the 576 isolates; Supplementary Fig. 4), preserving mutation types. We calculated 1,000 permutations for the null model (Supplementary Fig. 6). We limited our analysis to all coding sequences no longer than 2,000 amino acids, rather than weighting genes by their length.

### Measuring antibiotic resistance

We determined the resistance of an isolate as the highest level of antibiotic it could form a colony on solid agar while maintaining similar morphology to the colony on agar with no drug. The isolate library was pinned onto solid MacConkey II agar (BD 281810) poured in Nunc OmniTray Single-Well plates (VWR 62409-600). All isolates were pinned on a total of 16 plates (3 × 5+1): 5 concentrations for each of the three antibiotics (with technical duplicates), and one media-only plate. The antibiotic concentrations varied by twofold at each step: ceftazidime 100, 200, 400, 800 and 1,200 μg ml^−1^; ciprofloxacin 3.75, 7.5, 15, 30 and 60 μg ml^−1^; and tobramycin 37.5, 75, 150, 300 and 600 μg ml^−1^. After incubating at 37 °C for ∼60 h, all plates were imaged using an in-house system for taking pictures of plates with a Canon EOS Rebel T3i Digital SLR Camera. We analysed the images to determine for each isolate the highest concentration of drug at which growth was detected with a colony morphology similar to that of the no-drug plate.

### Comparing lineage distribution across sites with a well-mixed model

We permuted the site locations of all isolates in lineages A and S to construct the null model of a well-mixed environment. For each site, we calculated the ratio between isolates in lineage A and isolates in lineages A or S: A/(A+S). The distribution of observed ratios across all sites was compared with the expected using the χ^2^ goodness of fit.

### Spatial confinement

We calculated the fraction of isolate pairs separated by *d* or less SNPs that were also in the same site. This observed fraction was divided by the expected fraction calculated under a null model where isolates are randomly mixed between the sites, resulting in a likelihood measure. We designated this likelihood as our measure for spatial confinement *η*(*d*).


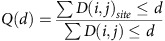



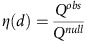


where *Q(d)*=the fraction of isolate pairs within genetic distance *d* that are in the same site and *η*(*d*)=measure for spatial confinement; compares the observed fraction of isolates in the same site with the expected fraction.

The spatial confinement measure *η*(*d*) was calculated at *d* for all integers from 0 to 60. The null model for *η* was constructed by permuting the site identity of isolates 1,000 times; statistics were calculated from these permutations.

### Estimating molecular clock and migration rate

Although a molecular clock is not known for *S. maltophilia* at the time of this work, we inferred a molecular clock from the mean root-to-dip distances over the 3-year infection that was 24 SNPs with 1 s.d. of 5 SNPs (Supplementary Fig. 13). This translated to 8 SNPs per year, with a s.d. of 1.7 SNPs. We used this approximation to convert 3 SNPs to 4.5 months.

### Synteny analysis of *merC* homologue

We used the online version of SyntTax^44^ to compare the synteny of *merC* homologues within the Xanthomonadales order.

### Data availability

The genomic sequence data for the 576 *S. maltophilia* isolates have been deposited in the Sequence Read Archive (SRA) database under accession code SRP090935. The authors declare that all other data supporting the findings of this study are available within the paper and its supplementary information files, or from the corresponding author on request.

## Additional information

**How to cite this article:** Chung, H. *et al*. Global and local selection acting on the pathogen *Stenotrophomonas maltophilia* in the human lung. *Nat. Commun*. **8**, 14078 doi: 10.1038/ncomms14078 (2017).

**Publisher's note:** Springer Nature remains neutral with regard to jurisdictional claims in published maps and institutional affiliations.

## Supplementary Material

Supplementary Information Supplementary Figures, Supplementary Tables and Supplementary References

Supplementary Data 1 Table of genotypes at polymorphic positions within the *S. maltophilia* population. Each row indicates a polymorphic position on the *S. maltophilia* Ab5555 genome, with the first number designating the contig and the second number indicating genomic position within that contig. Columns indicate the isolate.
