# Supplementary material for: Global and local selection acting on the pathogen Stenotrophomonas maltophilia in the human lung
Source: Nat Commun. 2017 Jan 19;8:14078. doi: 10.1038/ncomms14078 (PMC5253648; doi:10.1038/ncomms14078)
Supplement: Supplementary Information — Supplementary Figures, Supplementary Tables and Supplementary References [file ncomms14078-s1.pdf]

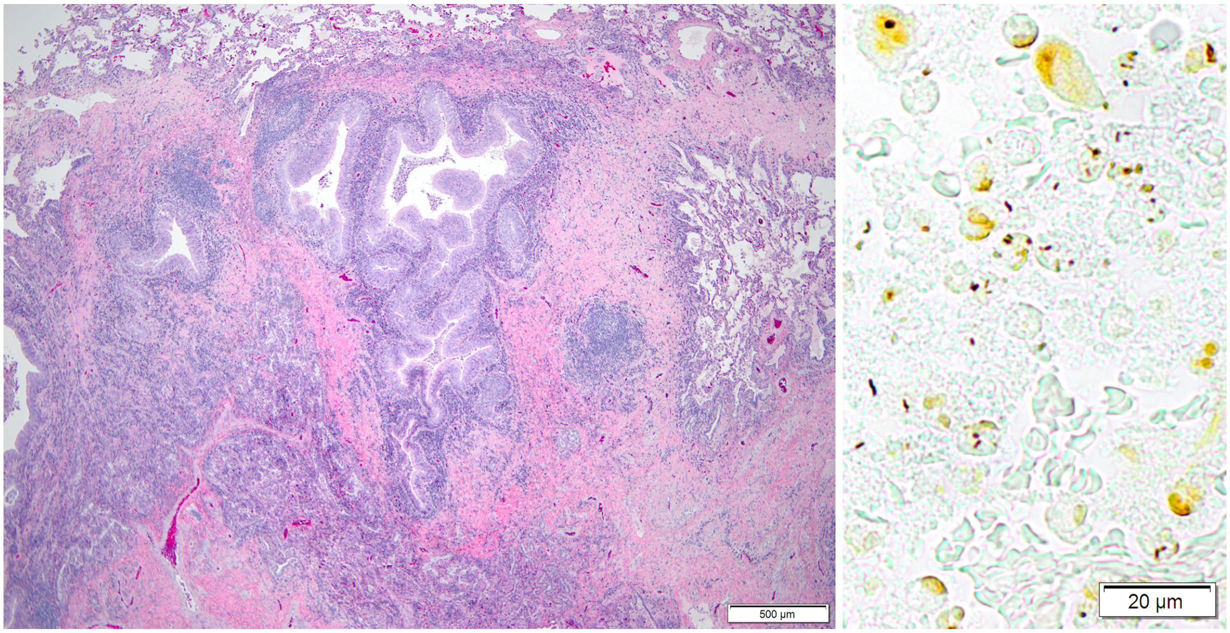

Supplementary Figure 1. Histologic examination of sample tissues confirmed a diversity of anatomic sites. A predominant finding was ectatic airways showing acute and chronic inflammation and periairway scarring as well as adjacent variably spared alveolated lung tissue (left, hematoxylin and eosin; original magnification, 40x). Special stains confirmed the presence of rod-shaped bacteria within different anatomic compartments of the lung (Steiner stain; original magnification, 600x).

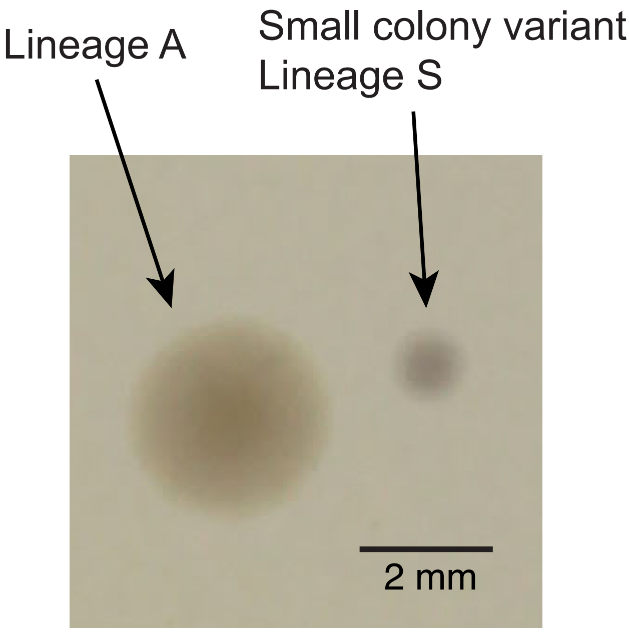

Supplementary Figure 2. Lineage S is a small colony variant. Image of isolates representing lineages A and S, respectively, shown after ~24h of growth on MacConkey agar.

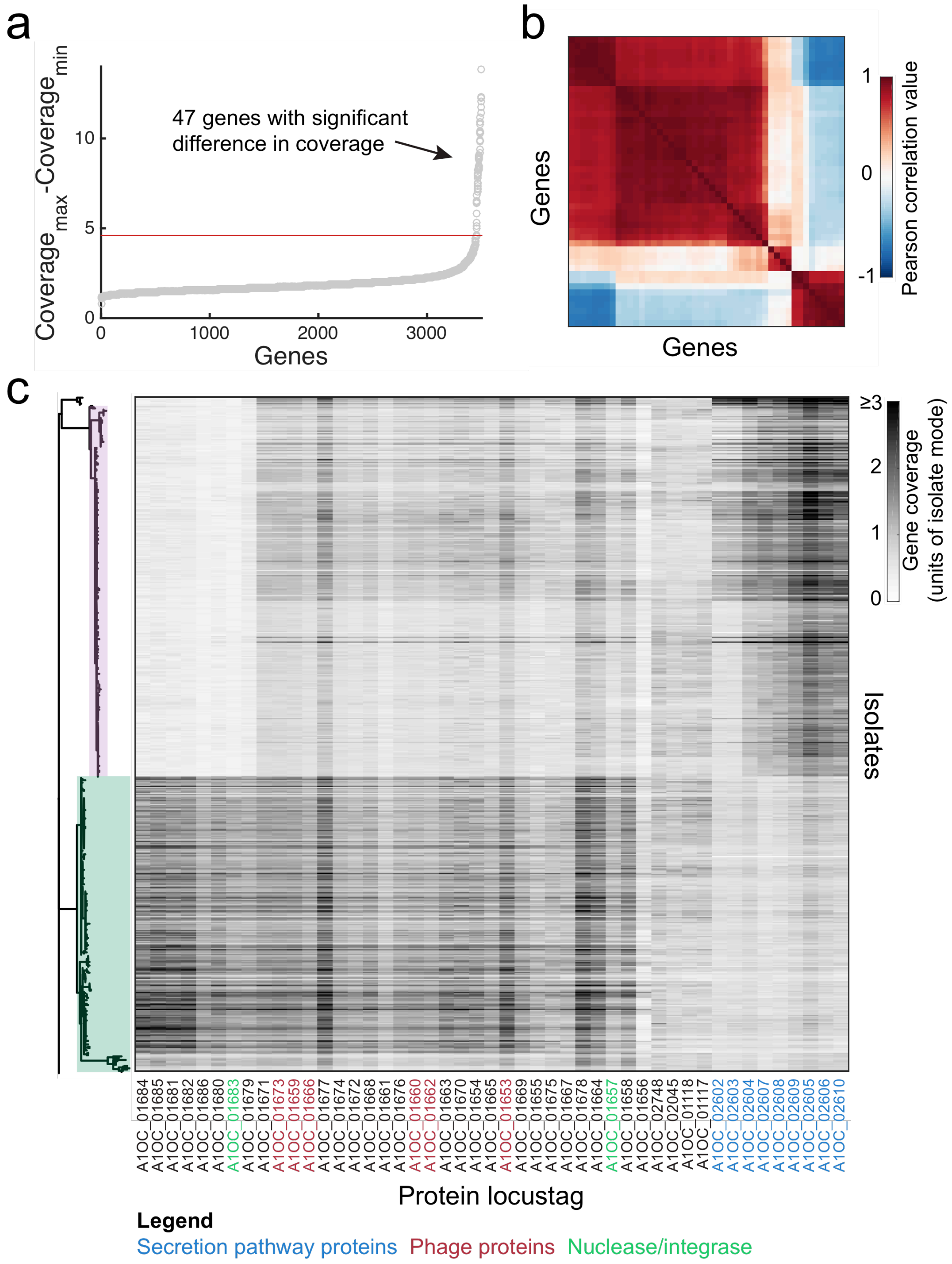

Supplementary Figure 3. Genes with copy number differences across isolates. (**a**) For each gene, we calculate the largest difference in coverage across isolates. We sorted the difference values and chose a cutoff threshold. (**b**) For all pairs of genes, we calculated the Pearson correlation of isolate coverage values. Clustering these correlation values showed 4 distinct blocks. (**c**) The copy number of 47 genes with significant differences is shown for each isolate aligned with the phylogeny. Further descriptions of genes are found in Supplementary Table 4.

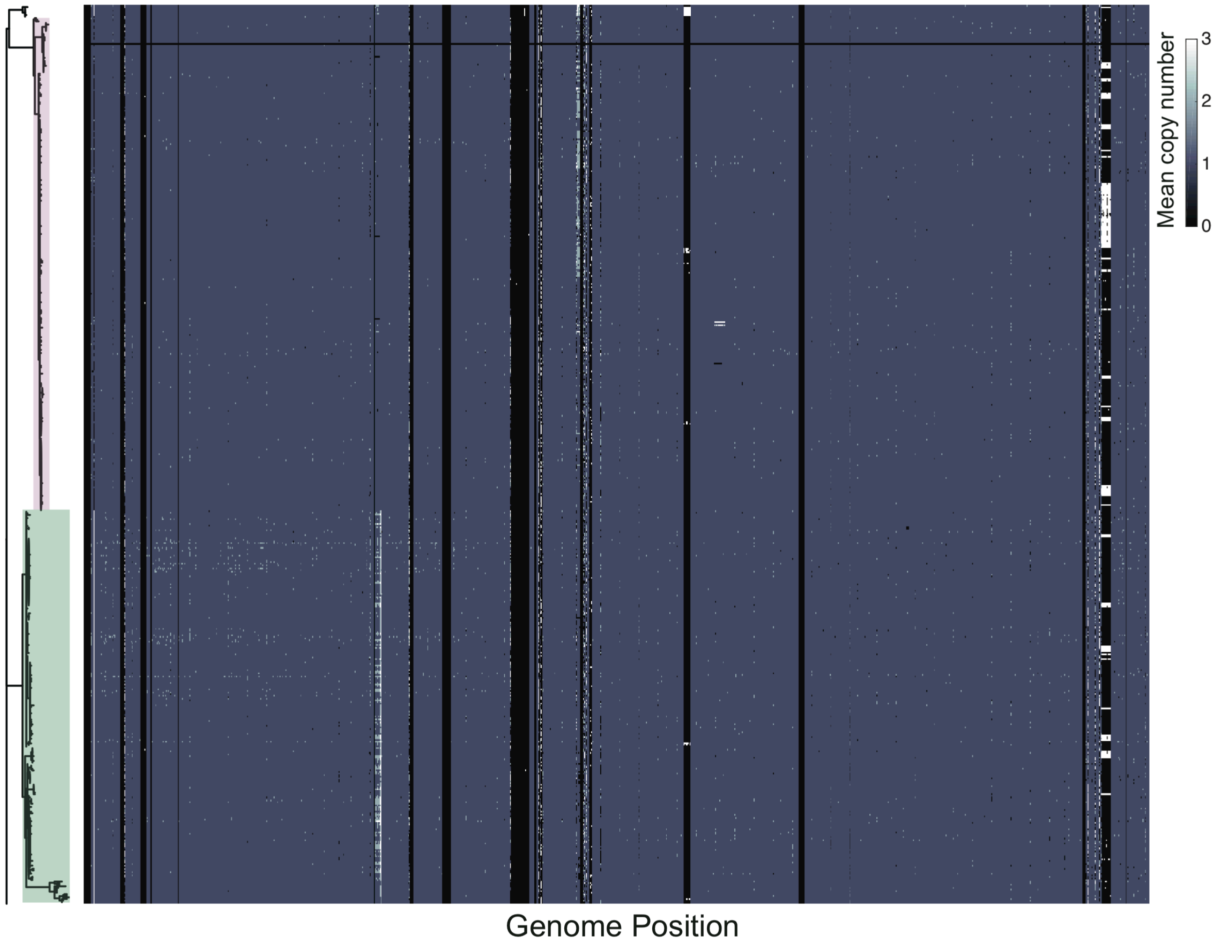

Supplementary Figure 4. Genome-wide coverage map of isolates against a reference strain indicates a lack of difference in genomic architecture, supporting that lineages are of the same strain. Coverage map across the Ab55555 reference genome and assembled contigs. Each row is an isolate corresponding to its position on the tree (left). The mean copy number at each genomic position is shown (Methods). At each position, coverage was divided by the mode coverage of each isolate, then divided by the median across isolates. Except for a few small regions with duplications, there is an absence of large-scale deletions or duplications between lineages.

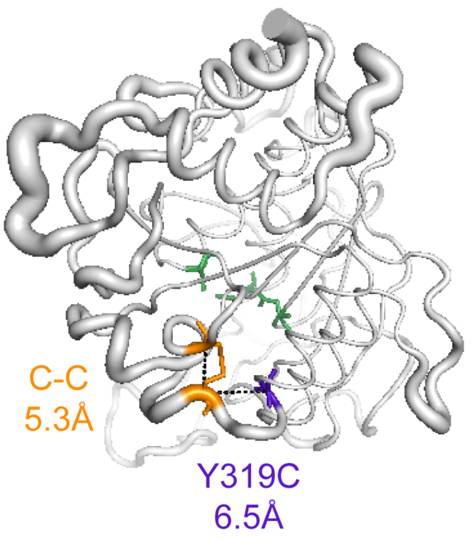

Supplementary Figure 5. Mutation in a serine protease (StmPr2 homolog) may disrupt a disulfide bridge in a disordered region of the protein. Mutations are mapped onto the crystal structure of a homolog, AprV2 (PDB ID: 3LPA; alignment E-value $8e^{-78}$)[1]. Residue Y319 of the serine protease, encoded by locustag A1OC_00723 on the *S. maltophilia* Ab55555 genome, maps to residue A187 in AprV2. The width of the protein cartoon is proportional to the B-factor, with thicker ribbons mapping to higher B-factor (less ordered). Residues in orange indicate existing cysteine residues that form a disulfide bridge; the distance between their alpha carbon atoms, 5.3Å, is indicated. The mutated residue (Y319C) is in purple, and the distance between the mutant and the nearest cysteine is 6.5Å, which is within the range to form a disulfide bond[2].

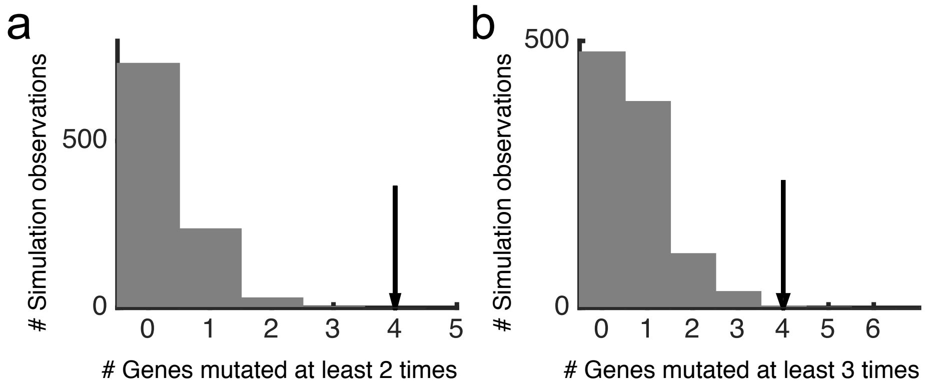

Supplementary Figure 6. Detecting genes with recurrent mutations that are under adaptive selection. (**a**) We observed 4 genes mutated at 2 or more positions by the 47 lineage-separating SNPs, which was significant compared to a null model where mutations were randomly distributed across the genome 1000 times ($P<10^{-3}$, bootstrapping a random allocation of mutations across the genome; Methods). (**b**) We observed 4 genes mutated at 3 or more positions by the 282 within-lineage SNPs, which was significant compared to a null model with 1000 permutations (P=0.004). The mutations found in these 8 genes are listed in Supplementary Tables 2 and 3.

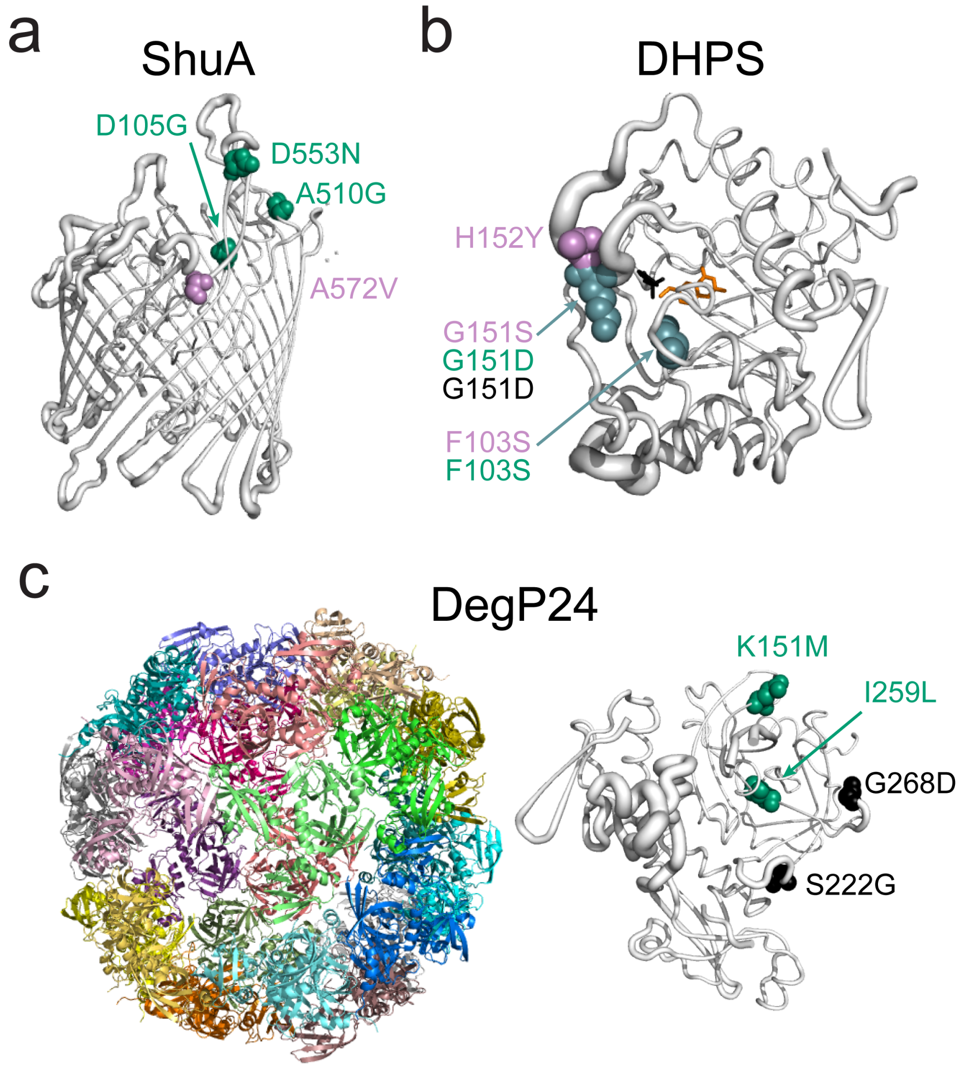

Supplementary Figure 7. Mutations occurring in genes under selection within the lineages mapped onto the crystal structures of homologs. Each mutation is colored by the lineage in which it occurred: pink (lineage A), teal (lineage S), black (lineage B). All amino acid positions of the mutations are with respect to the *S. maltophilia* protein. The width of the protein cartoon is proportional to the B-factor, with thicker ribbons mapping to higher B-factor (less ordered). (**a**) The mutated positions in the TonB-dependent heme/hemoglobin receptor protein are mapped onto the crystal structure of a homolog, ShuA (PDB ID: 3FHH)[3]. Residues mapped from the *S. maltophilia* Ab55555 homolog A1OC_00661 to ShuA as the following: D105 to D82, A510 to P441, D553 to D491, and A572 to A505. (**b**) The mutated positions of dihydropteroate synthase (DHPS) are mapped onto the crystal structure of its homolog in *E. coli* (PDB ID: 1AJ0)[4]. Residues mapped from locustag A1OC_01723 to DHPS as the following: F103 to S98, G151 to K146, and H152Y to T147. Two ligands, sulfanilamide and 6-hydroxymethyl-7,8-dihydropterin (DHP), are shown in black and orange, respectively. (**c**) The mutated positions of serine protease are mapped to a homolog, DegP24 (PDB ID: 3CS0)[5]. Left, DegP as a 24-mer with each DegP chain highlighted by a color. Right, a single DegP molecule with mutations marked. Residues mapped from locustag A1OC_03344 to DegP24 as the following: G268 to G233, I259 to I224, S222 to S188, and K151 to Q116.

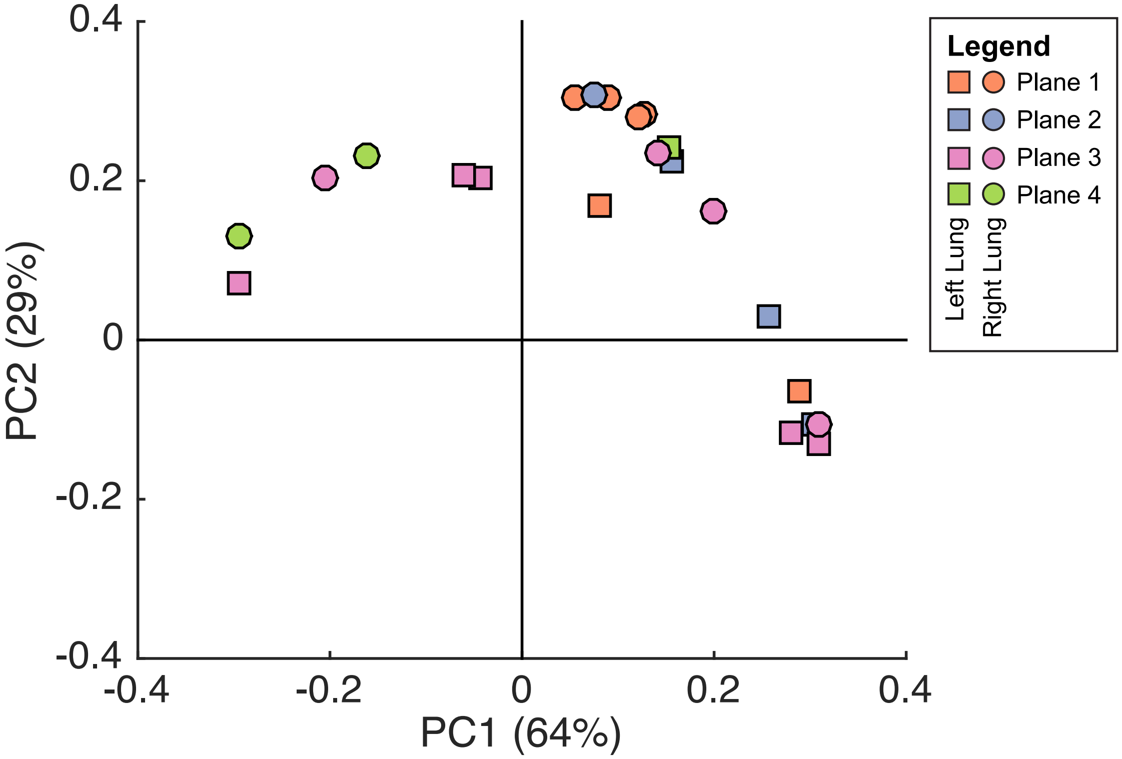

Supplementary Figure 8. Principal component analysis of beta diversity between site populations. Beta diversity between all pairwise site populations was calculated by weighted Unifrac[6]. Principal component analysis revealed that components 1 and 2 explain 64% and 29% of the variance, respectively. Each point represents PC1 and PC2 of a site population with the lung location and plane of sampling indicated (legend).

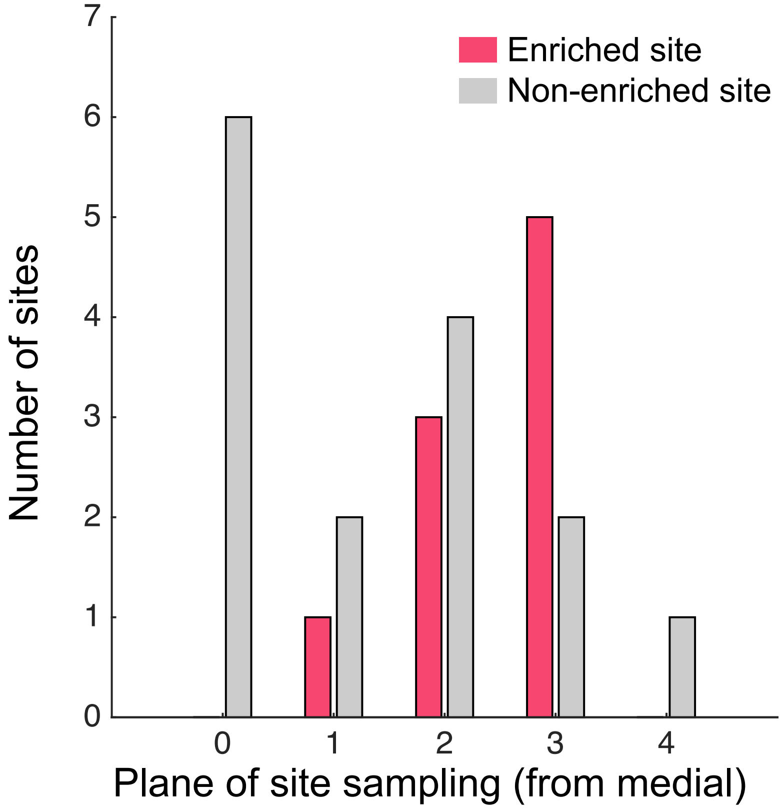

Supplementary Figure 9. Sites enriched with either lineage A or S correlate with being sampled from the peripheral planes of the lung. Sites were classified as lineage-enriched or not, with lineage-enriched sites defined as those that did not overlap with the null model distribution in Fig. 3b. The plane of sampling for each site is listed in Supplementary Table 1. Chi-squared test reveals that lineage-enriched sites are more significantly found in planes towards the periphery (P=0.03), suggesting the confinement of certain genotypes due to physical barriers to dispersion rather than selection.

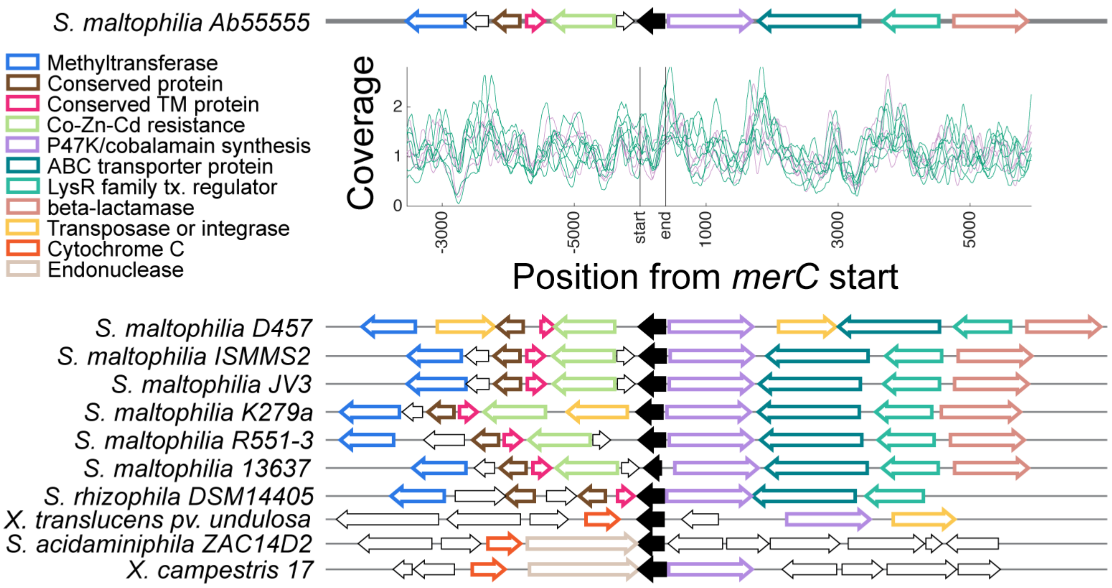

Supplementary Figure 10. Synteny analysis across closely related species of *S. maltophilia* show that the *merC* homolog is not found in the *mer* operon. The *merC* homolog (locustag A1OC_00410) from the *S. maltophilia* Ab55555 genome was compared to the Xanthomonadales order and analyzed for synteny via SyntTax[7]. Shown here are genome arrangements for top 10 strains that have the closest *merC* sequence to that of the Ab55555 strain. Coverage from 10 random isolates of the population are shown (normalized by the mode of each isolate), colored by their lineage membership (teal: lineage S, pink: lineage L). Filled black arrow indicates *merC*. Genes around *merC* that are part of conserved protein families are annotated.

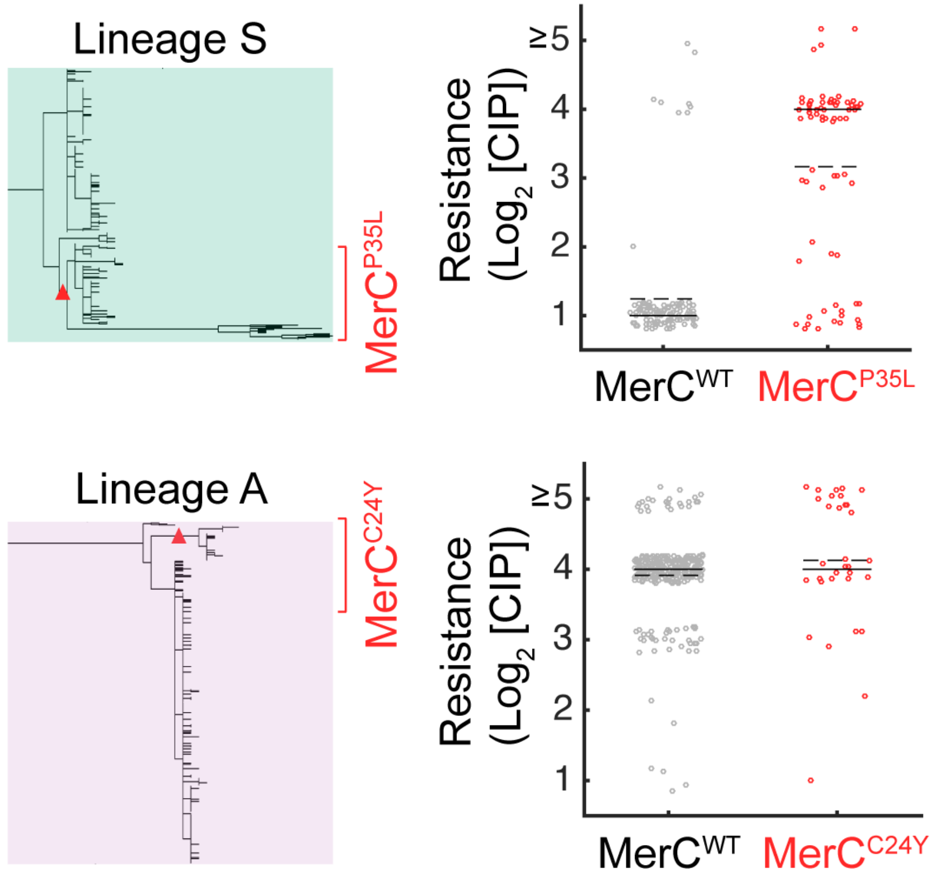

Supplementary Figure 11. MerC may play a role in ciprofloxacin resistance. Isolates with the P35L mutation in lineage S had a 8-fold increase in ciprofloxacin resistance compared to non-mutated isolates of lineage S (*P*=1.3e-19, Kolmogorov-Smirnov test) while the C24Y mutation in lineage A also led to an increase (*P*=0.0036, K-S test). However, linked mutations along the branch as well as subsequent mutations confound the precise effect of MerC mutations in ciprofloxacin resistance.

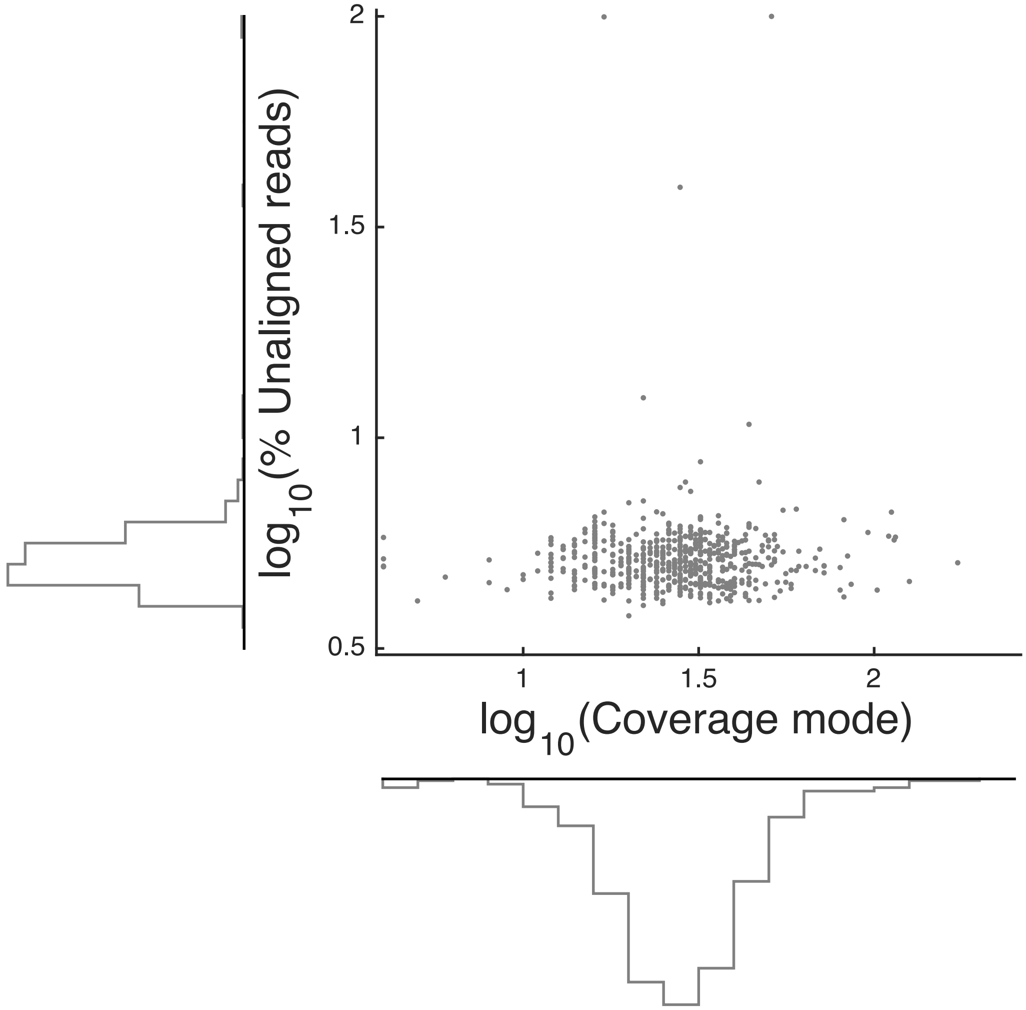

Supplementary Figure 12. Scatter plot of the coverage mode and percent unaligned reads of all 576 isolates. The marginal distribution is shown next to each axis. 98% of isolates had a coverage mode of 10 or higher, based on the alignment to the Ab55555 reference genome while 99% of isolates had less than 8% unaligned reads.

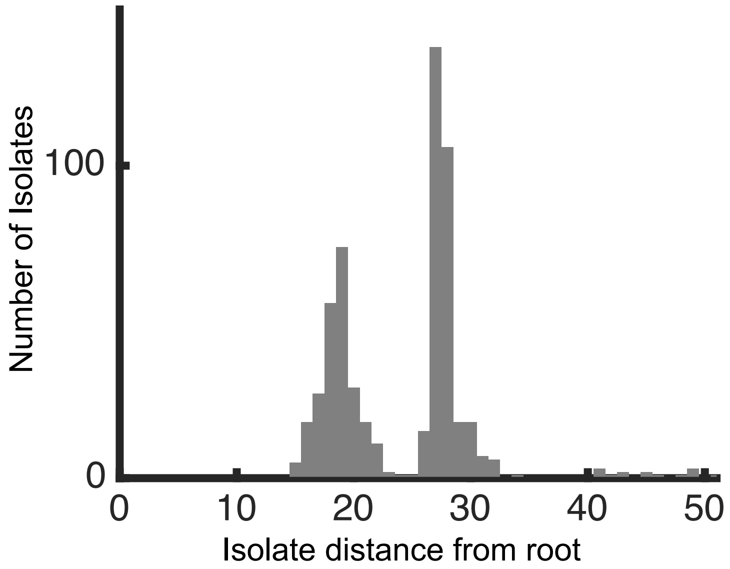

Supplementary Figure 13. Estimating a molecular clock from the distribution of root-to-tip distances. We calculated the SNP distance between the most recent common ancestor (MRCA) of the population and each isolate. The mean was 24 SNPs with a standard deviation of 5 SNPs. Dividing by the 3-year infection period yielded an estimate of 8 SNPs/year for the molecular clock.

Supplementary Table 1. Locations and descriptions of tissue samples. A total of 31 tissue samples were obtained. Samples that exhibited growth were assigned a site name; those that did not are in gray. Location is indicated as left lower lobe (LLL), left upper lobe (LUL), right lower lobe (RLL), right middle lobe (RML), or right upper lobe (RUL) unless otherwise specified. The parasagittal plane of cut from which site was sampled is indicated (0-4, medial to lateral). The degree of acute and chronic bronchopneumonia was assessed from histology slides. N/A, not available.

Site Name	Lung L/R	Plane (cuts)	Gross description	Location	Acute Inflamm	Chronic Inflamm
A	L	0	Bronchial margin	Left mainstem	none	moderate
	L	0	Hilar lymph node			
	L	0	Hilar lymph node			
B	L	1 thin	lower lobe bronchus	LLL	mild	moderate
	L	1 thin	small airway with pus for sample 6	LLL	mild	severe
C	L	1 thin	upper lobe bronchus	LUL	mild	moderate
	L	2 cuts	Pus from inside small airway	LLL		
D	L	2 cuts	inferior lower lobe lung tissue	LLL	mild	severe
E	L	2 cuts	peripheral lingula	LUL	severe	moderate
F	L	2 cuts	segmental airway	LLL	minimal	moderate
G	L	3 cuts	superior area of bronchiectasis (airway wall and airway contents)	LUL	mild	severe
H	L	3 cuts	superior area of bronchiectasis	LUL	severe	severe
I	L	3 cuts	inferior aspect of LUL	LUL	severe	severe
J	L	3 cuts	inferior bronchiectasis	LUL	mild	severe
	L	3 cuts	airway	LUL		
K	L	3 cuts	bronchiectatic airway inferior to I/J	LUL	moderate	severe
	L	3 cuts	Anterior-inferior with airway	LUL		
	L	4 cuts	subpleural lung tissue	LLL		
L	L	4 cuts	subpleural hemorrhage	LLL	N/A	N/A
M	R	0	bronchus	RML	minimal	mild
N	R	0	bronchus	RLL	none	moderate
O	R	0	bronchus	RUL	none	severe
P	R	0	hilar lymph node		N/A	N/A
Q	R	1 cut	segmental bronchus	RML	moderate	moderate
R	R	2 cuts	bronchiectatic bronchus	RUL	moderate	severe
S	R	2 cuts	bronchus	RUL	moderate	severe
	R	2 cuts	peripheral pale area	RLL		
T	R	2 cuts	brochiectatic airway (medium)	RML	moderate	severe
U	R	2 cuts	subpleural lung tissue	RLL	minimal	severe
	R	2 cuts	bronchiectatic bronchus	RML		
V	R	3 cuts	subpleural lung tissue (peripheral)	RLL	none	severe
W	R	3 cuts	N/A	RUL	N/A	N/A

Supplementary Table 2. Lineage-separating mutations. The locus tag, mutation type (N: non-synonymous, S: synonymous, P: promoter), position within the gene, and a description of the gene is listed for each mutation. We considered a sequence to be a promoter if it was within 150bp upstream of the start codon of a gene. Star (*) indicates a nonsense mutation. Subcellular localization ("Loc") was computationally inferred via PSORTb v.3.0[8] and Phobius[9], indicating cytoplasm (C), cytoplasmic membrane (CM), extracellular (E), transmembrane domain (TD), non cytoplasmic (NC), or unknown (U). A mutation found in an extracellular protein is highlighted in green. The four genes with recurrent mutations are highlighted in blue, and other genes implicated in antibiotic resistance in red.

**Locus**	**Type**	**Pos**	**Loc**	**Annotation**
A1OC_04203	S	P182	NC	efflux transporter, RND family, MFP subunit
A1OC_04203	S	A179	NC	efflux transporter, RND family, MFP subunit
A1OC_04443	S	T90	CM	diguanylate cyclase (GGDEF) domain protein
A1OC_04492	N	E178G	TD	protein tonB
A1OC_00345	N	N203T	NC	glycosyl hydrolase family, fibronectin type III domain (bglX)
A1OC_00372	N	R137G	U	putative pyrimidine metabolism (*rutE* homolog)
A1OC_00372	N	S147P	U	putative pyrimidine metabolism (*rutE* homolog)
A1OC_00483	N	N201D	C	transcriptional response regulator (RegX3/*mtrA* homolog)
A1OC_00484	N	S182L	CM	OmpR/PhoB 2CS response regulator
A1OC_00486	N	R12H	C	OmpR/PhoB 2CS response regulator
A1OC_00575	P			fimbrial adhesin protein
A1OC_00578	N	Q481*	OM	hemic uptake protein hemP
A1OC_00661	N	A572V	OM	TonB-dependent hemoglobin/transferrin/lactoferrin receptor family protein
A1OC_00662	N	Q189*	U	Haemin-degrading HemS.ChuX domain
A1OC_00668	N	G112R	CM	2CS Histidine Kinase
A1OC_00723	N	Y319C	E	extracellular serine protease (StmPr2 homolog)
A1OC_00795	N	S177A	C	3-deoxy-7-phosphoheptulonate synthase
A1OC_00823	N	D118N	C	amidophosphoribosyltransferase
A1OC_01179	N	S210L	CM	ubiquinol oxidase, subunit II
A1OC_01365	S	R368	C	DNA topoisomerase IV, A subunit
A1OC_01365	N	R368L	C	DNA topoisomerase IV, A subunit
A1OC_01365	N	Y81H	C	DNA topoisomerase IV, A subunit
A1OC_01460	N	S39C	C	lysine decarboxylase
A1OC_01736	P			LuxR family transcriptional regulator
A1OC_01775	N	P146A	C	glucose-6-phosphate isomerase
A1OC_01791	N	V42L	CM	succinate dehydrogenase flavoprotein subunit
A1OC_01820	N	T164M	C	LysR substrate binding domain
A1OC_01820	N	G90S	C	LysR substrate binding domain
A1OC_01979	P			cold-shock DNA binding protein

A1OC_01979	P			cold-shock DNA binding protein
A1OC_01989	P			RND family efflux transporter MFP subunit
A1OC_02190	S	A581	CM	ferrous iron transporter B
A1OC_02195	N	F236L	C	Enoyl-(Acyl carrier protein) reductase
A1OC_02227	N	P8L	C	chemotaxis response regulator protein-glutamate methylesterase 2
A1OC_02304	N	S86A	U	methyl-accepting chemotaxis protein
A1OC_02321	N	Y241*	C	PQQ domain (glucosyltransferase-I precursor)
A1OC_02854	S	H75	U	metal dependent phosphohydrolase
A1OC_02973	N	Q672R	C	NAD-glutamate dehydrogenase
A1OC_03002	N	D447N	C	dihydrolipoyl dehydrogenase
A1OC_03061	N	W379*	C	ribosomal large subunit pseudouridine synthase B
A1OC_03101	N	Q27*	U	surface antigen protein
A1OC_03332	N	P20S	C	hypoxanthine phosphoribosyltransferase
A1OC_03834	N	P326L	CM	hydrophobe/amphiphile efflux-1 (HAE1) family RND transporter
A1OC_03835	N	R272C	CM	efflux transporter, RND family, MFP subunit
A1OC_03858	N	D270N	C	pyridoxal-phosphate dependent lyase
A1OC_03884	P			dihydrolipoamide acetyltransferase
Contig_NODE_64	S	A22		glucose-1-dehydrogenase

Supplementary Table 3. Mutations of genes with recurrent mutations within lineages. The locus tag, mutation type (N: non-synonymous, S: synonymous, P: promoter), position within the gene (blank if promoter mutation), and a description of the gene is listed for each mutation. Subcellular localization ("Loc") was computationally inferred via PSORTb v.3.0[8] and Phobius[9], indicating cytoplasm (C), cytoplasmic membrane (CM), outer membrane (OM), or non cytoplasmic (NC).

Locus	Type	Pos	Loc	Annotation
A1OC_00410	N	P35L	CM	mercury resistance protein (*merC* homolog)
A1OC_00410	N	C24Y	CM	mercury resistance protein (*merC* homolog)
A1OC_00410	N	L16P	CM	mercury resistance protein (*merC* homolog)
A1OC_00661	N	D105G	OM	TonB-dependent heme/hemoglobin receptor (*shuA* homolog)
A1OC_00661	N	A510G	OM	TonB-dependent heme/hemoglobin receptor (*shuA* homolog)
A1OC_00661	N	D553N	OM	TonB-dependent heme/hemoglobin receptor (*shuA* homolog)
*A1OC_00661	N	A572V	OM	TonB-dependent heme/hemoglobin receptor (*shuA* homolog)
A1OC_01723	N	F103S	C	dihydropteroate synthase (DHPS)
A1OC_01723	N	G151S	C	dihydropteroate synthase (DHPS)
A1OC_01723	N	G151D	C	dihydropteroate synthase (DHPS)
A1OC_01723	N	H152Y	C	dihydropteroate synthase (DHPS)
A1OC_03344	N	G268D	NC	serine protease (*mucD/htrA* homolog)
A1OC_03344	N	I259L	NC	serine protease (*mucD/htrA* homolog)
A1OC_03344	N	S222G	NC	serine protease (*mucD/htrA* homolog)
A1OC_03344	N	K151M	NC	serine protease (*mucD/htrA* homolog)

*The *shuA* homolog was detected as a gene with recurrent mutations within the lineages, but it also happened to be mutated once along the parent branch of lineage A. Highlighted in red indicates this mutation along lineage A.

Supplementary Table 4. Genes that exhibit significant copy number difference between isolates. Annotations were obtained from UniProt[10] and Pfam[11].

Locus	Annotation
A1OC_01684	Hypothetical protein
A1OC_01685	Blue-light sensing BLUF domain
A1OC_01681	Uncharacterized protein
A1OC_01682	KAP P-loop domain protein
A1OC_01686	Vi polysaccharide biosynthesis protein VipA/TviB or UDP-N-acetyl-D-galactosamine dehydrogenase
A1OC_01680	Uncharacterized protein
A1OC_01683	integrase or recombinase (XerD homolog)
A1OC_01679	Uncharacterized protein
A1OC_01671	Uncharacterized protein
A1OC_01673	phage tail protein
A1OC_01659	terminase
A1OC_01666	HK97 gp10 family phage protein
A1OC_01677	Uncharacterized protein
A1OC_01674	Uncharacterized protein
A1OC_01672	Uncharacterized protein
A1OC_01668	Uncharacterized protein
A1OC_01661	Uncharacterized protein
A1OC_01676	Uncharacterized protein
A1OC_01660	HK97 family phage portal protein
A1OC_01662	HK97 family phage major capsid protein
A1OC_01663	Uncharacterized protein
A1OC_01670	Uncharacterized protein
A1OC_01654	Uncharacterized protein
A1OC_01665	Uncharacterized protein
A1OC_01653	phage lambda Rz-like lysis protein
A1OC_01669	Uncharacterized protein
A1OC_01655	Uncharacterized protein
A1OC_01675	Uncharacterized protein
A1OC_01667	Uncharacterized protein
A1OC_01678	Uncharacterized protein
A1OC_01664	Uncharacterized protein
A1OC_01657	HNH endonuclease family protein
A1OC_01658	Uncharacterized protein
A1OC_01656	Uncharacterized protein
A1OC_02748	Uncharacterized protein
A1OC_02045	Uncharacterized protein
A1OC_01118	Uncharacterized protein
A1OC_01117	Uncharacterized protein

A1OC_02602	General secretion pathway protein F
A1OC_02603	General secretory pathway protein E
A1OC_02604	General secretion pathway protein D
A1OC_02607	Type II secretion system protein K
A1OC_02608	General secretion pathway protein G
A1OC_02609	Uncharacterized protein
A1OC_02605	Uncharacterized protein
A1OC_02606	General secretion pathway protein L
A1OC_02610	Uncharacterized protein

Supplementary References

1. Kennan, R. M. *et al.* The Subtilisin-Like Protease AprV2 Is Required for Virulence and Uses a Novel Disulphide-Tethered Exosite to Bind Substrates. *PLoS Pathog* **6,** e1001210–12 (2010).
2. Schmidt, B. & Hogg, P. J. Search for allosteric disulfide bonds in NMR structures. *BMC Struct Biol* **7,** 49–12 (2007).
3. Cobessi, D., Meksem, A. & Brillet, K. Structure of the heme/hemoglobin outer membrane receptor ShuA from Shigella dysenteriae: Heme binding by an induced fit mechanism. *Proteins* **78,** 286–294 (2010).
4. Achari, A., Champness, J. N. & Bryant, P. K. Crystal structure of the anti-bacterial sulfonamide drug target dihydropteroate synthase. *Nat Struct Biol* **4,** 490–497 (1997).
5. Krojer, T. *et al.* Structural basis for the regulated protease and chaperone function of DegP. *Nature* **453,** 885–890 (2008).
6. Lozupone, C. A., Hamady, M., Kelley, S. T. & Knight, R. Quantitative and Qualitative β Diversity Measures Lead to Different Insights into Factors That Structure Microbial Communities. *Applied and Environmental Microbiology* **73,** 1576–1585 (2007).
7. Oberto, J. SyntTax: a web server linking synteny to prokaryotic taxonomy. *BMC Bioinformatics* **14,** 1–1 (2013).
8. Yu, N. Y. *et al.* PSORTb 3.0: improved protein subcellular localization prediction with refined localization subcategories and predictive capabilities for all prokaryotes. *Bioinformatics* **26,** 1608–1615 (2010).
9. Käll, L., Krogh, A. & Sonnhammer, E. L. L. A Combined Transmembrane Topology and Signal Peptide Prediction Method. *J. Mol. Biol.* **338,** 1027–1036 (2004).
10. Consortium, T. U. UniProt: a hub for protein information. *Nucl Acids Res* **43,** D204–D212 (2015).
11. Finn, R. D. *et al.* The Pfam protein families database: towards a more sustainable future. *Nucl Acids Res* **44,** D279–85 (2016).
